# Recurrent massive myxoid liposarcoma in retroperitoneum 10 years post initial operation

**DOI:** 10.1002/iju5.12115

**Published:** 2019-08-20

**Authors:** Takamasa Horiuchi, Shigekatsu Maekawa, Wataru Obara, Haruki Kume, Naoki Matsuoka

**Affiliations:** ^1^ Department of Urology The University of Tokyo Tokyo Japan; ^2^ Department of Urology Misato Central General Hospital Saitama Japan; ^3^ Department of Urology Iwate Medical University Iwate Japan

**Keywords:** follow‐up, liposarcoma, myxoid liposarcoma, recurrent tumor, soft tissue tumors

## Abstract

**Introduction:**

Myxoid liposarcoma tends to be a relatively good prognosis. Follow‐up is recommended once a year from the 5th year after surgery.

**Case presentation:**

A 48‐year‐old man had a massive retroperitoneal tumor, measuring approximately 20 cm. Resection of a huge myxoid liposarcoma had been performed on his back 10 years ago and the weight of the mass was 14 kg. The tumor was resected from the right kidney and its weight was 3.6 kg. Pathological examination showed myxoid liposarcoma and therefore, the tumor was diagnosed as recurrent. But after the resection, no recurrence or metastasis was observed.

**Conclusion:**

To our knowledge, the case presents the first report where both initial and recurrent tumors were massive and the time for recurrence was 10 years. Therefore, a follow‐up of myxoid liposarcoma might have to be done cautiously throughout the patient's life.

**Abbreviations & Acronyms**
CT: computed tomography
LP: liposarcoma
MLS: myxoid liposarcoma
MRI: magnetic resonance imaging

**Keynote message**

The term of LP follow‐up is recommended once a year after 5 years. In our case, no recurrence was found for 7 years after the first surgery, but the tumor relapsed after 10 years. Imaging follow‐up for LP had to be carried out throughout the patient's life.

## Introduction

Soft tissue sarcomas are unusual tumors and include 1.0–1.5% of all malignancies. The most frequent histological types are LP, leiomyosarcoma, and malignant fibrous histiocytoma. LP is characterized by malignancy of adipose tissue, accounts for 10–16% of all sarcomas, and is reported to be caused by unknown genetic alterations. It most commonly affects people in the age group of 50–70 years and is reported to have a slight male predominance.^1^ Diagnosis of LP requires CT or MRI, but diagnosis is often difficult. Therefore, tumor biopsy is usually required for clinical diagnosis. There are three histologic subtypes in LP: well and dedifferentiated (<50%), myxoid/round cell (<40%), and pleomorphic (10%). Myxoid/round cell LP typically occurs in younger individuals.^2^

The first‐line treatment for LP without metastasis is to radically resect the tumor. The 5‐year disease‐specific survival rate depends on following the pathological subtypes: 90–100% in well‐differentiated and/or dedifferentiated LP, 88% in myxoid/round cell LP, and 56% in pleomorphic LP.^3^

Here, we report the case of a patient with recurrent huge MLS in the retroperitoneum 10 years after the initial operation.

## Case presentation

**Figure 1:**
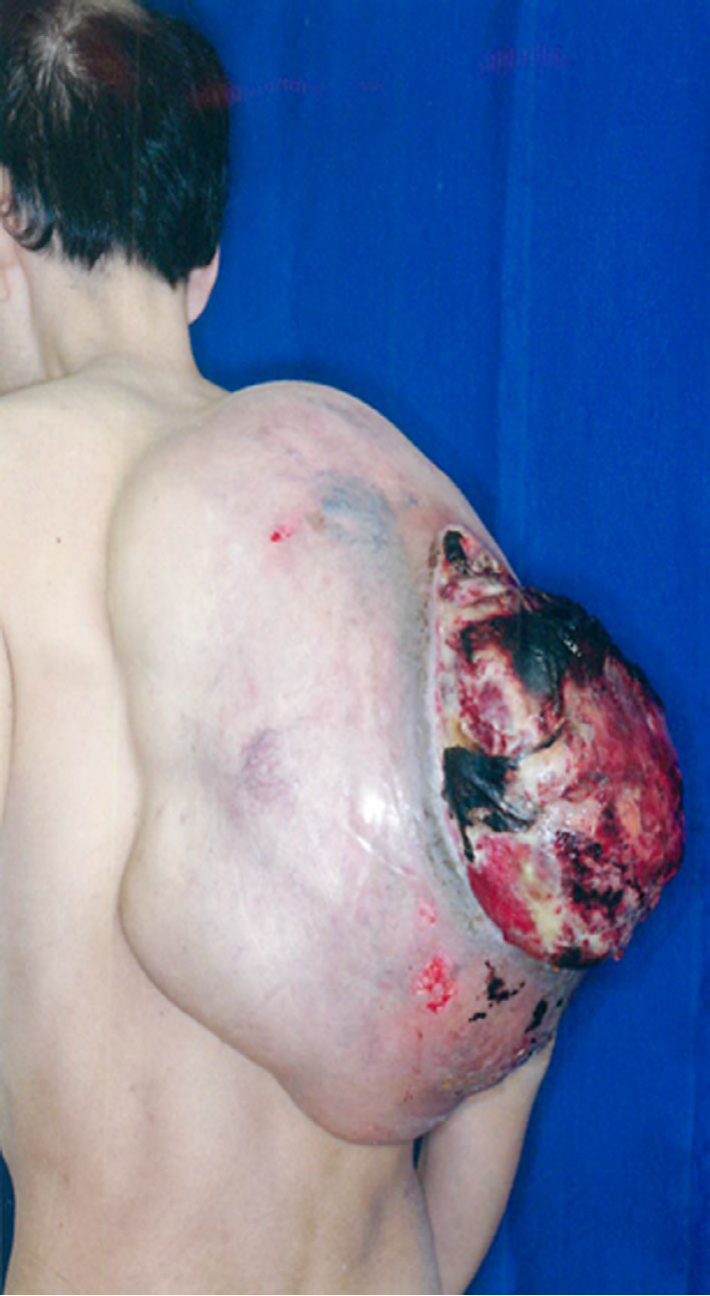
A huge tumor on the back measuring 44 × 30 cm.

**Figure 2:**
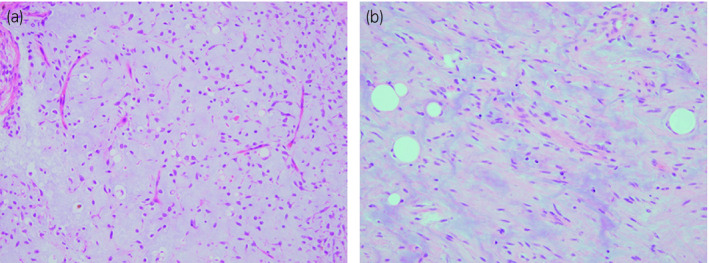
Pathological finding of hematoxylin and eosin staining. (a) H&E staining of back tumor which was resected in 2005 showing the MLS. (b) H&E staining of retroperitoneum tumor which was resected in 2015 showing the MLS.

**Figure 3:**
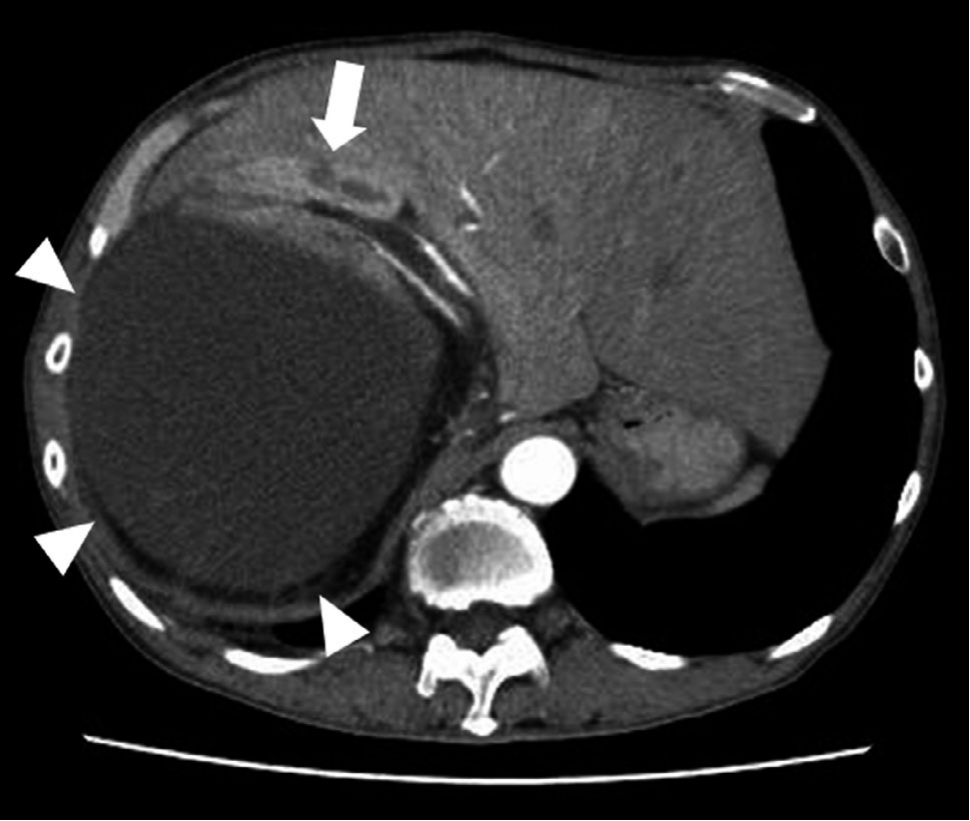
Abdominal CT scan showing the homogeneous enhancement of the tumor measuring >20 cm (white triangle), which compressed the right kidney (white arrow).

**Figure 4:**
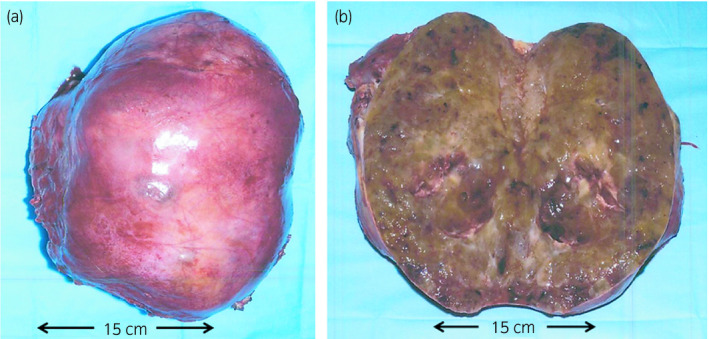
A tumor in the retroperitoneum measuring 23 × 16 cm. (a) The smooth external surface. (b) Cut surface yellowish, gelatinous and firm gray‐white tissue with areas of hemorrhage.

A 48‐year‐old man presented at our hospital with bleeding from a huge mass on his back in October 2005 (Fig. 1). Blood tests yielded minor leukocytosis and moderate anemia. He had noticed the growing mass on his back from 2000 but had not approached any hospital. After hospitalization, tumor biopsy was performed and the diagnosis was MLS. Post diagnosis, resection of a huge tumor on the back and local flap angioplasty were performed in November 2005. The size and weight of the resected tumor were 44 × 30 × 26 cm and 14 kg, respectively. The pathological diagnosis was MLS (Fig. 2a). As the surgical margins of the specimen were positive, he also received adjuvant chemoradiotherapy. Follow‐up CT was performed every year until 2011 and no evidence of recurrence or metastasis was observed. In August 2015, he presented at our hospital with a complaint of dehydration. Blood tests revealed acute kidney injury and immediate medical treatment was started. Approximately a 20‐cm retroperitoneal tumor was coincidentally observed in CT. He was referred to our department for treatment. Contrast‐enhanced CT revealed a retroperitoneal tumor sized approximately 20 cm without an evidence of metastasis (Fig. 3). A wide resection of retroperitoneal tumor combined with right kidney was performed in September 2015. The resected tumor measured 23 × 16 × 13.5 cm and weighed 3.6 kg (Fig. 4). The pathological diagnosis showed MLS (Fig. 2b) and was the similar to the tumor resected 10 years ago. A gene translocation could be detected neither initial nor recurrent tumor. The patient had no evidence of recurrence 12 months post operation.

## Discussion

We report a case of large recurrent MLS over 10 years. MLS usually occurs in the deep‐seated soft tissues of the extremities with the most common occurring sites being the thigh (13–60%) and retroperitoneum (10–36%) and rarely the back.^4^ In our case, the initial MLS was on the back. A translocation of FUS and DDIT3 (CHOP) genes is found around over 95% of myxoid and round cell LPs.^2^

Complete surgical resection with negative microscopic margins is the only curative treatment for MLS. LP tends to form a pseudo‐capsule with an indistinct border.^5^ Therefore, an aggressive surgical technique with en‐bloc multi‐organ resection is required, as performed in this study. It was recently reported that a 5‐year local recurrence‐free survival after only surgery and surgery combined with radiotherapy was 23–54% and 40–62%, respectively. The 5‐year rate of overall survival was 33–49% and 48–64%, respectively. In our case, the surgical microscopic margins were positive during the initial operation, and thus, we additionally conducted adjuvant chemoradiotherapy. After resection, metastasis and recurrence did not occur for 5 years.

MLS primarily metastasizes to extrapulmonary sites including bones such as spines and other soft tissues such as retroperitoneum, in contrast to the other subtypes of LP which mainly metastasize to the lung.^6^ The percentage of MLS metastasis ranges from 14% to 32%,^7^ and its prognostic factors have been previously reported. Factors strongly associated with a poor prognosis include age (>45 years), tumor size (>10 cm), high histological grade, and percentage of round cell differentiation (>5%).^8^ The occurrence of a round cell component is the most significant factor for local recurrence, metastasis, and survival.^7^ The risk of local recurrence is 3.4 times greater if a tumor consists of more than 5% round cells. Also, patients with <5% round cells are at a greater risk of poor prognosis compared to pure MLS, 0% round cell population.^9^ In this case, we expected a good prognosis as the population of round cells was <1% and 0% on the back and in the retroperitoneal tumor, respectively.

The European Society for Medical Oncology recommends a follow‐up every 3–4 months for the first 2–3 years, then twice a year for up to 5 years, and annually thereafter for patients with intermediate or high‐grade LP. For patients with low‐grade LP, a follow‐up every 4–6 months for 3–5 years and then annually is recommended. Standard follow‐up practice should include the following: imaging (CT/MRI) with a focus on local recurrence and routine chest X‐ray to exclude pulmonary metastases or CT to detect metastases.^10^ In our case, no recurrence occurred after 7 years, but tumor relapsed after 10 years. Therefore, our case indicated that all LP patients, irrespective of risk grade, have to receive imaging follow‐up throughout their life. The main limitation of this study is that it is a single case report; therefore, larger sample size is required to establish an updated follow‐up protocol for LP patients.

To our knowledge, this is the first case that the size of both initial and recurrent tumors was massive, 14 kg and 3.6 kg, respectively, and time for recurrence was 10 years.

## Conflict of interest

The authors declare no conflict of interest.
